# Mini-review: Serum immunoglobulins and MASLD

**DOI:** 10.3389/fimmu.2025.1655560

**Published:** 2025-08-15

**Authors:** Ke-qian Chen, Wen-jing Cao, Shu-zhi Wang, Wei Li

**Affiliations:** ^1^ Department of Clinical Pharmacy, Xiangtan Central Hospital (The Affiliated Hospital of Hunan University), Xiangtan, China; ^2^ Institute of Pharmacy and Pharmacology, School of Pharmaceutical Sciences, University of South China, Hengyang, China

**Keywords:** serum immunoglobulins, serum biomarker, MASLD, NASH, fibrosis

## Abstract

With the development of society and the economy, metabolic dysfunction-associated steatotic liver disease (MASLD) has become a major chronic disease in contemporary society. Finding a safe, effective, and economical diagnostic method is essential for the prevention of MASLD. Serum immunoglobulin is a protein produced by the B cells after the body is stimulated by an external antigen or pathogen. It is very interesting and valuable to explore the relationship between serum immunoglobulins and MASLD. Unfortunately, only a small number of studies have explored the relationship between serum immunoglobulins and MASLD. Therefore, we review the research progress of serum immunoglobulins in MASLD. At the same time, we also discuss the shortcomings of these studies. We hope this review will provide experience and reference for the prevention of MASLD in the future.

## Introduction

Metabolic dysfunction-associated steatotic liver disease (MASLD) is a clinicopathologic syndrome characterized by excessive fat deposition in liver cells, which is caused by no-alcohol and other specific liver damaging factors (1). In recent decades, with the increase of sedentary lifestyles and Western dietary patterns, MASLD has gradually become a growing public health problem (2). Therefore, how to diagnose MASLD and judge the development of MASLD is very significant. Studies have shown that the stages of MASLD include metabolic-dysfunction associated steatotic liver (MASL), metabolic dysfunction-associated steatohepatitis (MASH), liver fibrosis, and cirrhosis (3). In 1998, British scholars proposed the “two-hit” theory for the first time in the world (4). The theory suggests that the first shock is liver lipid deposition and insulin resistance, and the second shock is oxidative stress. However, current research indicates that the pathogenesis of MASLD is quite complex. MASLD may be the result of a combination of factors, including genetic factors, nutritional factors, metabolic conditions, drug use, and surgical conditions (5). Because MASLD is a complex and progressive disease, it is necessary to establish a reliable diagnostic method. Currently, liver biopsy is considered the gold standard for the diagnosis of MASLD (6). However, it is an invasive method with serious limitations. Liver biopsy is not suitable for all MASLD patients. Other diagnostic methods also have limitations: For example, traditional ultrasonography largely depends on the experience of the ultrasound examiner, which is highly subjective and may result in missed diagnosis of MASLD patients. CT scans can detect lipid accumulation in liver cells, but cannot determine the stage of liver fibrosis. Serum immunoglobulin is a protein produced by the B cells after the body is stimulated by an external antigen or pathogen (7). As an important part of human humoral immunity, they participate in the resistance to viral and bacterial infections by binding antigens or pathogens. Earlier studies found that serum immunoglobulin (IgA) concentrations were commonly elevated in alcoholic liver disease (ALD) patients (8). MASLD and ALD have common pathophysiological features and histological features, suggesting that they may have a common pathogenic mechanism. In addition, elevated serum immunoglobulins have been observed in autoimmune hepatitis (raised IgG) and primary biliary cirrhosis (raised IgM) (9, 10). B cells, which produce immunoglobulins, are also the most common immune cells in the liver. Emerging evidence has demonstrated that B cell activation is an important event in the progression of MASLD (11). In liver biopsies of MASLD patients, the accumulation of B cells was positively correlated with the MASLD activity score (11). Serum B-cell activating factor was also elevated in MASH patients (12). During MASH, B cells can aggravate insulin resistance, hepatic inflammation, and liver damage by producing pathogenic antibodies (lgG) (13). As mentioned above, we speculate that the development of MASLD may also be related to serum immunoglobulins. Immunoglobulins may play an important role in the diagnosis of MASLD. Unfortunately, only a small number of studies have explored the relationship between serum immunoglobulins and MASLD. Therefore, we review the research progress of serum immunoglobulins in MASLD. At the same time, we also discuss the shortcomings of these studies. We hope this review will provide experience and reference for the prevention of MASLD in the future.

## MASLD and immunoglobulins

It is well known that the liver is a complex and important organ in the human body. Exploring the relationship between immunoglobulins and liver is also of great significance for us to understand the relationship between immunoglobulins and MASLD. Yu Lei et al. examined the expression of immunoglobulins in the liver of humans and rats (14). They found that hepatocytes can synthesize lgG, IgA and IgM, but not IgD and IgE. In addition to normal hepatocytes, malignant hepatocytes can also produce immunoglobulins. These immunoglobulins play an important role in the liver and other organs. For example, IgA in the liver can protect other organs from pathogens through portal circulation (15). As a growth factor, IgG has significant effects on the proliferation, apoptosis and migration of hepatocytes (16). The body’s immune system is mainly defended by two types of lymphocytes (T cells and B cells) (17). B cells are derived from stem cells in bone marrow. Its main function is to produce antibodies (18). T cells are derived from lymphoid stem cells in the thymus. On the one hand, it can directly kill target cells. On the other hand, it can assist or inhibit the production of antibodies (19). It is well known that antibodies are the body’s immune products against pathogen infection. In essence, it is a globulin with immune function. For this reason, antibodies are also called immunoglobulins. Studies have shown that immunoglobulins are present in serum, body fluids, and some cell membranes (20). Immunoglobulin molecule is composed of two identical light chains (L chain) and two identical heavy chains (H chain). L chain and H chain are connected by disulfide bonds to form a tetrapeptide chain molecule (monomer of Immunoglobulin molecule), which is the basic structure of immunoglobulin molecule. According to the structure of H chain constant region, immunoglobulins are divided into five types: IgG, IgA, IgM, IgD, and IgE (21).

## MASLD and IgG

Immunoglobulin G (IgG) is the main component of serum immunoglobulins, accounting for about 75% of the total immunoglobulins. As the most important antibody in the primary immune response, IgG is mainly synthesized and secreted by plasma cells in the spleen and lymph nodes. When the human body is infected by pathogens, IgG appears late and has the longest half-life (22). Meanwhile, IgG is the only immunoglobulin that can cross the placental barrier (23). It plays a protective role in the infection of young mammals and newborns. At present, many studies have explored the relationship between lgG and immunoglobulins. In MASH mice, oral administration of lgG can alleviate chronic inflammation and liver injury (24). Shao Mei Sun et al. performed a cross-sectional study to assess the relationship between serum immunoglobulin concentrations and MASLD (25). The study evaluated 11261 Chinese adults recruited from January 2010 to December 2015 via multiple logistic regression analysis. They found that the prevalence of MASLD was negatively associated with IgG. Karrar A found that MASLD patients had significantly lower levels of IgG when compared with controls. IgG may play a protective effect role in the pathogenesis of MASLD (26). Paradoxically, some studies have put forward opposite results. Osman HA et al. also performed a cross-sectional study to assess the role of serum immunoglobulins in MASLD. Their study involved 100 MASLD patients and 100 healthy volunteers. They found that IgG were increased in MASLD patients. IgG may be involved in the progression of MASLD (27). In a prospective multi-center cohort study, De Roza MA et al. found that IgG was elevated in MASH patients. The mechanism may be related to IgG production induced by oxidative stress in MASH (28). Besides the MASH, Albano et al. also found that IgG was significantly elevated in the advanced fibrosis stage of MASLD patients (29). We think there are some flaws in the above results. The sample sizes of some studies are small and the diagnosis of MASLD is based on an unblinded histological interpretation of the local pathological team. Therefore, the factor of interobserver bias cannot be ruled out. Nevertheless, these studies still suggest that IgG may be involved in the pathogenesis of MASLD. Therefore, a large amount of research is still needed in the future to explore the relationship between MASLD and lgG.

## MASLD and IgA

Studies have shown that IgA can be divided into secretory IgA(SIgA)and serum IgA. The content of serum IgA is second only to IgG, accounting for about 15% of serum immunoglobulins (30). However, serum IgA does not show immune function in serum. The SIgA is a dimer. As the main antibody against infection in the mucosal area of the body. SIgA mainly exists in the mucosa of respiratory tract, digestive tract, and urethra (31). When the synthesis of SIgA is insufficient, the respiratory tract and digestive tract of newborns are more susceptible to infection. Studies have shown that IgA cannot cross the placenta (32). Although IgA is not present in neonatal serum, SIgA can be obtained from breast milk (32). The cross-sectional study by Shao Mei Sun and Osman HA indicated that the prevalence of MASLD was positively correlated with IgA. Tomita K et al. want to determine whether serum IgA could diagnose fibrosis in MASLD patients (33). They studied 151 biopsy-proven Japanese MASLD patients and 22 healthy control subjects between April 2002 and December 2010 at Keio University Hospital. The results suggest that serum IgA was a valid independent predictor for assessing pre-cirrhosis progression of MASH. Stuart McPherson et al. want to evaluate serum immunoglobulin levels in biopsy-proven MASLD patients (34). They studied 285 patients who attended a tertiary fatty liver clinic between 1999 and 2009. These patients were tested for serum immunoglobulins within 6 months of liver biopsy. They found that serum IgA levels were elevated in MASLD patients. Serum IgA levels can be an independent predictor of advanced fibrosis. Maleki I et al. tested serum IgA levels in 50 biopsy-proven MASLD patients (35). They found that the higher the IgA levels, the higher the degree of fibrosis. IgA shows its value in distinguishing fibrosis in MASLD patients. In a large cohort of MASLD patients (941 patients), Elias E et al. also found that about 25% of MASLD patients had elevated serum IgA levels (36). Paradoxically, in a large pediatric cohort, Mouzaki M et al. found that serum IgA levels were elevated in only 4% of patients (37). Serum IgA is not an optimal biomarker for MASLD children. We think that the limitation of this study include its retrospective nature. In addition, their cohort was predominantly non-Hispanic and reflected the demographics of their regional pediatric MASLD population. A large number of studies have shown that intestinal dysbiosis is related to the severity of human MASLD. Interestingly, the mucosal immune system may further affect the function of serum IgA (38). When the mucosal barrier is damaged, Kupffer cells in the liver trigger the expression of FcaRI. Then IgA induces Kupffer cells to phagocytize fcari (39). Therefore, intestinal dysbiosis may partially explain the relationship between MASLD and IgA levels.

## MASLD and IgM

Immunoglobulin M (IgM) is the largest immunoglobulin with molecular weight, accounting for about 10% of the total immunoglobulins (40). As a pentamer, IgM is also known as macroglobulin and is mainly secreted by plasma cells in the spleen and lymph nodes (40). Studies have shown that IgM is mainly present in the blood and mucosal surface and divided into IgMl and IgM2 two subtypes (41). When the body is infected, the first antibody that appears is IgM. Therefore, the detection of IgM levels can be used as an early diagnostic indicator of infectious diseases. Unfortunately, IgM has the disadvantages of high molecular weight and not easy to penetrate blood vessels. At present, only a small number of studies have explored the association between lgM and MASLD. Sun SM et al. found that the prevalence of MASLD was negatively associated with IgM (25). Hendrikx et al. found that IgM titers against oxidation-specific epitopes (OSE) were less in subjects who had MASLD than controls in two cohorts from USA (42). These results suggest that the lower level of IgM antibodies against oxidized lipids might be specific for MASLD.

## MASLD and IgD

The molecular structure of IgD is very similar to IgG, accounting for about 1% of the total immunoglobulins. Studies have shown that IgD is unstable and easily cleaved by enzymes (43). However, the exact function of IgD in serum remains unclear. Its main physiological function may be to prevent immune tolerance and some hypersensitivity reactions (44). Regrettably, only one study explored the relationship between lgD and MASLD. Moreover, this study did not observe any significant changes in the level of secreted IgD in the plasma of MASLD mice (45). Therefore, we speculate that there is no association between lgD and MASLD. A large amount of research is still needed in the future to explore the relationship between MASLD and lgD.

## MASLD and IgE

IgE is the lowest immunoglobulin in the serum. Current studies have shown that IgE is mainly distributed in the respiratory tract and intestinal mucosa (46). Its main function is related to inhibiting parasitic infection (47). At present, only a small number of studies have explored the association between lgE and MASLD. Sun SM et al. detected a higher level of serum IgE in MASLD participants. However, no significant relationship between MASLD and IgE was detected after adjustment for confounding factors. It is worth noting that a slightly elevated IgE concentration may be associated with the advanced fibrosis in MASLD. Therefore, it is necessary to further study the role of IgE in MASLD.

The spectrum of MASLD includes MASL, MASH, and fibrosis. Only 20% of MASL patients will progress to MASH, and among these MASH patients, 20% will develop fibrosis (48).

## Discussion

**Figure 1:**
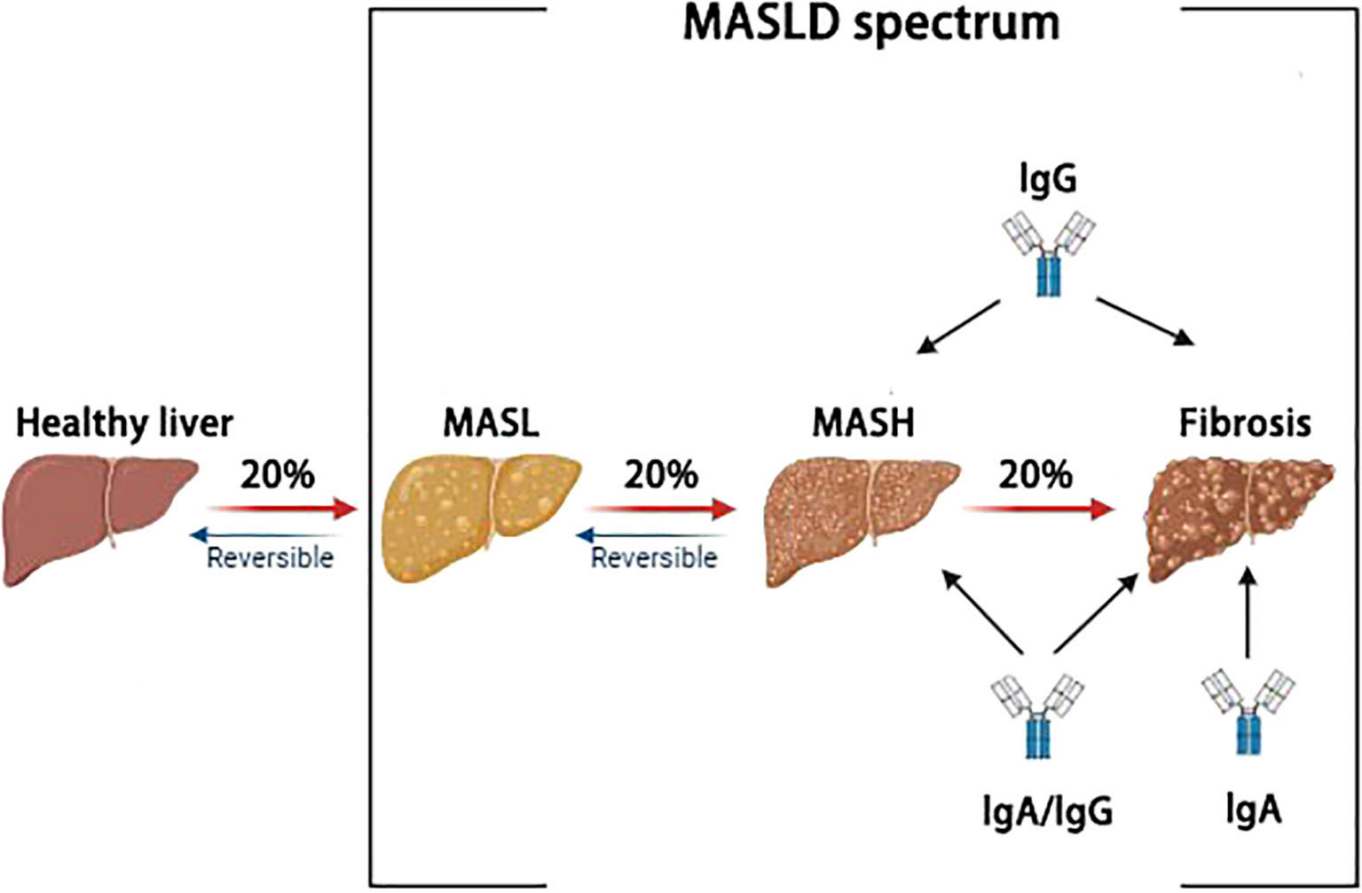
The role of immunoglobulins in MASLD.

The above results indicate that IgG and IgA play an important role in MASLD (**Table 1**, **Figure 1**). Immunoglobulin type conversion is a biological mechanism that enables B cells to transform the antibodies they produce from one type to another. Among immunoglobulins, IgA and IgG can be converted from IgM (7). Meanwhile, IgA and IgG are also the main forces of the body’s anti-infection defense. The main difference between them lies in their functions: IgA promotes the secretion of secretory peptides through mucosal surfaces, while IgG does not. Additionally, IgA and IgG differ in their transport methods, complement activation, and roles in passive immunity (49). Similarly, IgA/IgG ratio also plays an important role in MASLD. Ichikawa T et al. evaluated the clinical significance of IgA in 478 new patients who visited the Outpatient Clinic of Nagasaki Harbor Medical Center. They found that the IgA/IgG level was highest in patients with ALD, followed by those with MASLD and non-SLD (50).

**Table 1:** A summary of reports on IgG, IgA, IgA/IgG, and MASLD.

Population(MASLD)	IgA	IgG	IgA/IgG	Ref.
4598	↑	↓	\	(25)
98	—	↓	\	(26)
100	↑	↑	\	(27)
261	\	↑	\	(28)
167	\	↑	\	(29)
285	↑	—	\	(34)
50	↑	\	\	(35)
941	↑	↑	\	(36)
478	↑	↑	↑	(50)

↑, Increase ↓, Decrease —, No change \, No report.

With the development of economy and the change of life style, MASLD has become a major chronic disease in contemporary society. Finding a safe, effective, and economical diagnostic method is essential for the prevention of MASLD. The liver is one of the most important internal organs of the human body. On the one hand, the liver plays an important role in endocrine and exocrine activities. On the other hand, the liver also plays a key role in the immune system. Many studies have shown that immunotherapy can improve liver damage. Therefore, exploring the relationship between serum immunoglobulins and MASLD is very interesting. At present, compared with other serum biomarkers for diagnosing MASLD, serum immunoglobulins have advantages and importance. On the one hand, the relative molecular mass and content of serum immunoglobulins are higher than other serum biomarkers (CK-18, miRNA), and the detection accuracy is relatively high (51). On the other hand, serum immunoglobulins are more accurate than other serum biomarkers (adipocytokines) in diagnosing the liver fibrosis stage of MASLD (51). Immunoglobulin as a serum biomarker also has many limitations: Firstly, only a few clinical studies investigated serum immunoglobulins in MASLD patients, and a large number of studies are still needed in the future to explore its accuracy. Secondly, serum immunoglobulins levels may vary due to various diseases, which may reduce the specificity in diagnosing certain conditions such as MASLD. Thirdly, serum immunoglobulins levels may vary with different stages of MASLD, which also reduce the specificity in diagnosing certain conditions such as MASLD. Moreover, there are conflicting results in these studies. We think that the conflicting results may be due to the different stages of MASLD or different methods of serum immunoglobulin measurement. These clinical studies should be improved. First, serum immunoglobulin levels should be assessed in a larger cohort of patients with MASLD. Meanwhile, different ethnic groups are recommended in the study. Second, clinical studies should follow patients to determine whether serum immunoglobulin levels predict the subsequent course of MASLD. Third, clinical studies should increase the number of biopsy available for analysis. Meanwhile, it is also important to eliminate other unidentified factors that lead to changes of serum immunoglobulin levels.

In addition to improving clinical studies, two other questions need to be addressed. The first question is that the cause of changes of serum immunoglobulins in MASLD patients is unknown. What are the factors that affect the changes of serum immunoglobulins in MASLD patients? Do these factors also affect serum immunoglobulins in other liver diseases? The second question is whether the changes of serum immunoglobulins are involved in the pathogenesis of MASLD. Up to now, the pathological mechanism of MASLD has not been fully elucidated. Therefore, understanding the pathogenesis of MASLD has become a research hotspot in the field of liver and metabolism. Current studies have shown that the pathogenesis of MASLD includes insulin resistance, lipotoxicity, mitochondrial dysfunction, endoplasmic reticulum stress, inflammation, nutritional factors, gut microbiota, and genetic factors (52–54). Whether serum immunoglobulins can play an important role in these pathogenesis is an interesting research direction. In conclusion, serum immunoglobulin analysis is a cheap and non-invasive diagnostic method. Further studies are still needed to explore the relationship between serum immunoglobulins and MASLD.
